# analysis outbreak methicillin-resistant s. aureus (MRSA) in our unit. description of the control measures

**DOI:** 10.1186/2197-425X-3-S1-A133

**Published:** 2015-10-01

**Authors:** R Fernández Fernández, M Herreros Gonzalo, J Granados Ortega, L Camacho Peinado, A Iburrun Larralde, F Alba García, MÁ Taberna Izquierdo, F Árbol Linde

**Affiliations:** Hospital Nuestra Señora del Prado, Unidad Cuidados Intensivos, Talavera de la Reina, Spain; Hospital Nuestra Señora del Prado, Medicina Preventiva y Salud Pública, Talavera de la Reina, Spain

## Objectives

Analyze the outbreak of MRSA locating possible causes, seeking carriers, describe the control measures carried out and analyze the impact of control measures in a general hospital. Evaluate the outcome.

## Methods

Prospective descriptive analysis of patients admitted to the Intensive Care Unit (ICU)with positive microbiological results for MRSA in 2013.

The work was done with the services of Microbiology and Preventive Medicine and Public Health. We made with Preventive Medicine some guidelines for outbreak control:

Insulation Contact: patient in an individual box signaling in front of the box

should be placed a poster explaining the type of isolation and characteristics.

Hand hygiene: before and after each patient contact. use 10% povidone iodine, chlorhexidine 4%.Use of disposable gloves and disposable gowns long sleeve (must be removed before leaving the room).

Decolonization treatment: hygiene will be held daily with chlorhexidine gluconate 4% or 10% povidone iodine.

Topic: only in patients with nasal colonization. Application of mupirocin ointment in paraffin base every eight hours for five days.

Systemic: exceptional indication, complementing the topical treatment in special situations: two new cases in ICU over a period of two weeks: Doxycycline 100 mg / 12 h + oral Rifampicin 600 mg / 24 h for 7 days.

Control samples: it will be sampling of nasal swabs, axillary and inguinal exudate and skin lesions weekly until three consecutive weeks.

## Results

From 11 to 17 July of 2013, we found 4 patients with microbiological resultspositive for MRSA. 3 of them were in ICU and 1 of them had been in ICU some days before. As in the previous six months (January-June 2013) we had a total of 3 patients with a positive culture for MRSA, we could consider that we were facing an outbreak. Until September 26th, when the outbreak is considered closed, we had 131 episodes of ICU admission, of these, 14 patients (8 women and 6 men) had positive samples for MRSA. The cumulative incidence is 11% of patients who were admitted during this period.Samples were positive in 8 cases and in 6 cases, clinical samples. Of these 6, 4 cases were also positive surveillance cultures. There was 1 death among the 14 cases, 1/14; 7.1%. The study of carriers starts on August 8. Of 93 workers, complete the nasal screening 91 of them. And three positive cases (3/91 = 3.2%), which became negative after treatment with nasal mupirocin are detected.

The outbreak is considered complete at 28 September.

## Conclusions

Active surveillance in UCI has to be initiated in patients at risk and all patients when the rate of MRSA is high, in which case they must implement preventive measures to eradicate it. Our measures have been effective in controlling our outbreak. ICU staff had 3 carriers, the possibility of studying prevalence of MRSA carriers among staff in the ICU should be considered.

## Grant Acknowledgment

Preventive Department

